# A Novel Transformer Architecture for Scalable Perovskite Thin-Film Detection

**DOI:** 10.3390/mi17030314

**Published:** 2026-02-28

**Authors:** Mengke Li, Hongling Li, Yuyu Shi, Yanfang Meng

**Affiliations:** 1 Department of Artificial Intelligence, Jiangsu Normal University Kewen College, Xuzhou 221132, China; 2 Department of Electrical and Intelligent Manufacturing, Jiangsu Normal University Kewen College, Xuzhou 221132, China; 3 School of Mechanical Engineering, Jiangsu University, Zhenjiang 212013, China

**Keywords:** transformer architecture deep learning, scalable, perovskite thin-film detection

## Abstract

The further development of scalable fabrication for perovskite solar cells has been considerably constrained by strong process variability and the lack of a reliable real-time predictive mechanism during the thin-film formation process. Existing machine learning-based methods are incapable of capturing the inherent multi-stage kinetic characteristics and uncertainties of the perovskite crystallization process, as they rely on deterministic point prediction models and flatten time-series signals into static features, which necessitates more advanced modeling strategies. To address these challenges, an in situ process monitoring and predictive modeling framework based on a lightweight probabilistic Transformer is proposed for the scalable preparation of perovskite thin films. The strategically designed inputs, consisting of time-resolved photoluminescence (PL) and diffuse reflectance imaging signals acquired during the vacuum quenching process, enable the model to directly learn the conditional probability distribution of the final device performance metrics. Rather than producing a single predicted value, this method enables the explicit quantification of prediction uncertainty, providing statistical support for uncertainty-aware process assessment. Leveraging its advantages over feed-forward neural networks and traditional tree-based machine learning methods, the proposed Transformer architecture effectively captures the staged and non-stationary kinetic features of thin-film formation. Consequently, it exhibits higher robustness and superior uncertainty calibration capability during the early-stage prediction phase. The results demonstrate that the probabilistic Transformer-based modeling paradigm provides a viable pathway toward uncertainty-aware, data-driven process evaluation in perovskite manufacturing. This framework extends its application beyond perovskite photovoltaic device fabrication, providing a generalizable modeling strategy for real-time predictive assessment in the preparation of other complex materials governed by irreversible stochastic dynamics.

## 1. Introduction

Extensive concern has been focused on the organic–inorganic hybrid perovskite (OIHP) materials, attributed to their tunable bandgaps, large optical absorption coefficient, high charge carriers mobility, and long exciton diffusion length [1,2,3,4,5,6]. However, significant challenges remain regarding the considerable variations and stochasticity in the crystallization pathway of perovskite films and the final device performance even at the identical nominal process parameters conditions, posing significant obstacles in industrial manufacturing [7,8].

The perovskite photoelectric conversion efficiency (PCE) quantifies the probability that an incident photon leads to the excitation and subsequent collection of a charge carrier. It is defined as the average number of carriers successfully harvested per incident photon and is determined by

$$EQE\left(\%\right)=\frac{R_{\lambda }hc}{e\lambda }$$

where $R_{\lambda }$ represents the photoresponsivity and *λ* denotes the incident wavelength. The power conversion efficiency (PCE) of solar cells is also closely correlated with $R_{\lambda }$, and $R_{\lambda }$ is linked to the band tail states, characterized by the Urbach energy [9].

The band tail width $E_{u}$ reflects the degree of disorder within the material.


$$E_{u}={\left(\frac{dln(PL)}{dE}\right)}^{-1}$$

Processing parameters such as annealing temperature, anti-solvent dripping time, and additive concentration directly affect $E_{u}$ and $R_{\lambda }$ thereby influencing the EQE [9]. Addressing this challenge spurs the implementation of machine learning methods to extract features from time-resolved photoluminescence, reflectance, or scattering images, thereby enabling the early assessment of film quality, compositional ratios, and device efficiency. This demonstrates significant potential for reducing experimental trial-and-error costs and accelerating process development. However, the above methods often show insufficiencies for dealing with blurry boundaries and low-contrast regions, frequently leading to the misclassification of lead iodide clusters or the erroneous merging of adjacent grains [10], embodying three fundamental limitations. First, most approaches formulate the prediction task as a deterministic regression or classification problem, merely providing a deterministic output while overlooking the inherent uncertainty prevalent in the perovskite crystallization process [11]. In fact, the ultimate goal of in situ process monitoring is not limited to “predict device performance earlier,” but to extend to make rational decisions during a stage where the process can still be adjusted, thereby reducing the risk of failure and improving overall yield. In this context, models relying solely on deterministic point predictions are insufficient to support reliable real-time predictive assessment, especially when different process pathways exhibit similar expected performance but significantly different risk profiles. Building on this understanding, this paper proposes probabilistic modeling for the parameters during the process of the scalable fabrication of perovskites. Rather than relying on fully connected layers, which are limited in modeling sequential dependencies [12], a Transformer-based architecture for perovskite process monitoring prediction is proposed here, yielding superior accuracy. Taking time-resolved photoluminescence and diffuse reflectance imaging signals collected during the vacuum quenching process as inputs, a lightweight Transformer architecture is applied to explicitly model the temporal structure of the thin-film formation process effectively, and directly learns the conditional probability distribution of device performance metrics. Employing a distributional modeling approach, the model delivers the expected performance value coupled with a quantified measure of its prediction uncertainty, thereby laying the groundwork for uncertainty-aware process assessment.

More importantly, in practical manufacturing, the reliability of predictions carries identical weight to the associated risk of failure; the reliability of predictions and the associated risk of failure is as important as, if not more critical than, the predicted mean value itself. Second, many models flatten in situ time-series signals into static feature inputs, thereby discarding the physically meaningful phasic and non-stationary temporal structure inherent in thin-film formation, which constrains the model’s ability to characterize complex kinetic processes. Third, current research primarily focuses on prediction accuracy per se, with inadequate exploration into how prediction results can be translated into uncertainty-informed process assessment to better support manufacturing evaluation. Furthermore, this paper incorporates predictive uncertainty into the in situ analysis framework, extending predictive modeling toward an uncertainty-aware process assessment paradigm. Comparative analysis to traditional deterministic models on experimental results demonstrates the proposed method’s higher predictive robustness and more reliable uncertainty calibration capabilities in the early stages of the process. It also possesses the ability to distinguish between process scenarios with similar predicted mean values but significantly different risk levels.

The main contributions of this work can be summarized as follows:

(1) A lightweight probabilistic Transformer-based method for in situ time-series modeling is proposed.
(2) Device performance uncertainty is explicitly quantified through distributed forecasting, with systematic evaluation of its physical consistency and statistical reliability.
(3) Predictive uncertainty is incorporated into an uncertainty-aware process evaluation framework, offering new insights for intelligent perovskite manufacturing.

## 2. Related Works

### 2.1. Machine Learning for Augmented Process Monitoring of Scalable Perovskite Thin-Film

Extensive concern has been focused on machine learning-enhanced approaches for controlling the morphology of perovskite thin films, enabling effective monitoring, prediction, understanding, and ultimately, well-controlled film formation [13,14,15]. ML-aided PSC design encompasses three distinct stages: compositional screening, material fabrication and stability analysis, and full device development and testing, or feature engineering, model selection, and model evaluation.

At the full device level, the research scope expands to encompass the remaining constituent layers, including the electron transport layer (ETL) and hole transport layer (HTL). MacLeod et al. recently demonstrated a self-driving laboratory capable of autonomously selecting optimal fabrication parameters for spiro-OMeTAD, a widely used HTL in perovskite solar cells (PSCs) [16]. Regarding the above instance, characterizing device stability under simulated environmental stressors and standard operating conditions is of ever-increasing importance. These evaluations often yield results that differ from those obtained for the isolated perovskite layer alone, primarily due to interfacial effects such as charge carrier recombination and contact resistance at the layer interfaces [17].

A hierarchical CNN was employed to predict the lattice constant and octahedral tilt angle for ABX_3_ perovskites and consequently made features as the input for a second CNN to predict the material bandgap [18]. Model input features were elemental and structural descriptors including ionization energies, electron affinities, and tolerance factors. Machine learning (ML) models can leverage historical data to predict the future performance of perovskite materials and devices under various environmental stressors, such as temperature, bias voltage, humidity, light, and oxygen exposure [19]. Studies on machine learning-based load forecasting and time-series prediction continue to emerge. This is achieved by leveraging these techniques to uncover correlations between in situ monitoring data—specifically, time-resolved imaging of photoluminescence and diffuse reflectance—and key perovskite material parameters, as well as the performance metrics of solar cells [20,21].

### 2.2. Deep Learning for Augmented Process Monitoring of Scalable Perovskite Thin Film

The relentless drive toward next-generation flexible electronics, soft robotics, and the Internet of Things (IoT) has created a critically high demand for high-performance perovskite devices. Deep learning, evolved from machine learning, represents a subsequent development in the field [22,23,24,25]. The mapping between the input and output is accomplished through two distinct computational stages: forward propagation and backward propagation [26].

Forward propagation involves feeding an input sample into the network and computing its output value. This process entails successively applying operations such as convolution, activation functions, and fully connected layers. Conversely, backward propagation begins with calculating the error (or loss) between the network’s predicted output from the forward pass and the sample’s true label. This error is then propagated backward through the network to determine the contribution of each layer to the overall error (i.e., gradient information). The calculated gradients are subsequently used to adjust the network’s parameters (weights and biases) via an optimization algorithm, a process iteratively repeated until the network converges or a predefined termination condition is met. During forward propagation, the training samples are fed into the network with initialized layer parameters. Through layer-by-layer computation, the network generates the output corresponding to the input sample under the current parameters, thereby completing one forward pass. In classification tasks, the final output vector typically represents the probability distribution of the sample belonging to each predefined class. The prerequisite for implementing this data-driven characterization approach is the availability of experimental in situ data to quantitatively assess thin-film quality, thereby establishing one-to-one mapping relationships between in situ data and quality metrics through deep learning model training [27,28,29]. For example, Laufer et al. put forward a deep learning-enhanced perovskite metrology for quality upgrades by monitoring material composition, predicting device performance, and generating recommendations for in situ process control [26]. They applied in situ photoluminescence and diffuse reflectance imaging during the vacuum quenching process—specifically, during the formation of the perovskite layer by blade coating.

Deep learning is widely employed for optimizing the crystal structure of perovskite thin films and for screening and predicting the performance of their modified materials [10,30,31]. For instance, in *Hirshfeld* surface analysis, modified computational protocols are introduced, which are specifically tailored to address subtle yet significant distinctions within inorganic crystals. The two-dimensional *Hirshfeld* surface fingerprint demonstrates a strong capability as a rich “database,” acting as an informative source that encompasses complex relationships between chemical bonding and bond geometry characteristics in perovskites.

Deep autoregressive model (DeepAR), a powerful tool for probabilistic forecasting, adopts a sequence-to-sequence architecture with identical encoder and decoder components to estimate the future probability distribution of time series. Following a similar paradigm, Ng et al. proposed a multilayer perceptron-based model for estimating *Gaussian* parameters to predict surgical case durations [32]. Subsequently, Wen et al. developed the Multi-Quantile Recurrent Neural Network (MQ-RNN), which improves the stability and performance of the encoder–decoder architecture by its sequence-to-sequence structure and an enhanced piecewise sequence training scheme, and the MQ-RNN delivers probabilistic multi-step forecasts [33]. This line of research has continued to evolve in recent years, with Rangapuram et al. proposing *DeepState*, a deep state-space model that integrates state-space modeling with deep learning, maintaining both data efficiency and interpretability while effectively learning complex patterns directly from raw data [33,34]. In 2022, Xiao et al. put forward a probabilistic short-term load forecasting method, examining error information from point predictions and incorporating the Bootstrap method to estimate confidence intervals [35]. Specially, representing as a seminal deep learning architecture, the Transformer has revolutionized Natural Language Processing (NLP) and serves as the core of modern Large Language Models (LLMs). Characterized by its sole reliance on the attention mechanism instead of recurrent structures, it captures dependencies across all positions in a sequence simultaneously [36]. This allows for efficient parallel training and robust modeling of long-range dependencies, overcoming the key limitations of previous approaches like RNNs. The strong expressive power of Transformer-based encoders enables the generation of probabilistic predictions [37].

## 3. Methodology

### 3.1. Problem Definition and Overall Framework

The performance of perovskite solar cells strongly depends on their dynamic evolution behavior during the fabrication process [38]. During perovskite vacuum quenching, grain coalescence, defect passivation and phase stabilization process in later stages and early PL intensity alone cannot completely correlate with final electronic quality. Time-resolved photoluminescence (TRPL): Distinguishing surface and bulk recombination. The origins can be categorized into the following aspects:

(1) Multi-exponential decay and stretched exponential fitting, which included three aspects.

① In the early stages of vacuum quenching, the PL decay typically exhibits multi-exponential behavior, which originates from spatial heterogeneity—a broad distribution of local carrier lifetimes across different regions. It is recommended to use a stretched exponential function for fitting:


$$I\left(t\right)=I_{0}e^{-{\left(\frac{t}{\tau }\right)}^{\beta }}$$

Here, the stretching exponent β (0 < β ≤ 1) quantifies the degree of heterogeneity in the local lifetime distribution. A β value closer to 1 indicates a more uniform film, whereas a smaller β suggests the presence of numerous non-radiative recombination centers. If β is initially low but increases in the later stage, it provides direct evidence that grain fusion and defect passivation have improved the film uniformity [39].

② Excitation Wavelength-Dependent Analysis

Short-wavelength excitation (high absorption coefficient): The generation of charge carriers near the surface region makes the PL decay sensitive to surface defects.

Long-wavelength excitation (low absorption coefficient): Charge carriers are generated in the bulk, reflecting the quality of the bulk material. If surface passivation takes place, the decay lifetime under short-wavelength excitation will be significantly prolonged [39].

③ Excitation Intensity-Dependent Analysis

The variable excitation intensity TRPL measurements provide the possibilities to distinguish between monomolecular recombination (defect-assisted) and bimolecular recombination (radiative recombination). Under low excitation intensity, the carrier concentration is low, and defect trapping dominates. The PLQY increases with excitation intensity due to the gradual saturation of defects. Under high excitation intensity, Auger recombination may be triggered, leading to a decrease in PLQY.

Validation strategy: Conduct excitation intensity-dependent PL measurements at different stages of the vacuum quenching process. In the later stages, the slope of PLQY increase with excitation intensity becomes shallower and the saturation plateau reaches a higher level; this indicates a reduction in defect density [40].

(2) Quasi-Fermi Level Splitting (QFLS) Measurement

Quasi-fermi level splitting (QFLS) measurement directly reflects the chemical potential of photogenerated carriers and serves as a critical parameter for evaluating voltage losses:


$$E_{F}=E_{F,n}-E_{F,p}$$

Validation approach: Calculate QFLS through absolute PL intensity measurements. An increase in QFLS during the later stages of vacuum quenching indicates reduced non-radiative recombination losses, which will ultimately lead to higher open-circuit voltage (Voc) in the final device [41].

The photoluminescence (PL) decay kinetics are closely related to the excitation wavelength.

The in situ monitoring signals derived from fabrication reflect complex physical processes such as material crystallization, phase transitions, and defect formation, serving as important sources of information for characterizing the final device performance [29]. However, in actual fabrication and testing processes, complete process signals can only be obtained after the fabrication process is completed [42]. In the early stages of fabrication, the model can only access partial in situ monitoring signals up to the current moment, while the dynamic evolution of subsequent processes remains unknown. This kind of time-series observable data pose a significant impediment to directly applying them to early-stage performance prediction tasks.

**Figure 1:**
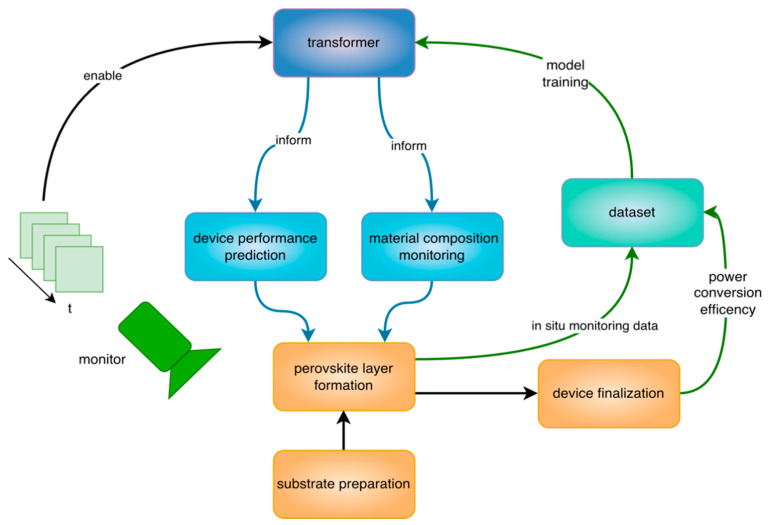
Overview of the proposed early-stage multi-task learning framework for perovskite fabrication.

Figure 1 illustrates the multi-task modeling framework. During the perovskite thin-film formation process, multi-channel time-series signals—consisting of diffuse reflectance signals (ND) and three photoluminescence bands (LP725, LP780, and SP775)—are synchronously collected using an in situ optical system to characterize the dynamic evolution of the film. Based on this framework, this study primarily investigates the feasibility of jointly modeling key device attributes given only partial in situ monitoring signals from the early stages of the fabrication process.

Formally, we assume that $s_{1:t}=\{s_{1},s_{2},\ldots ,s_{t}\}$, $s_{1:t}=\{s_{1},s_{2},\ldots ,s_{t}\}$ which denotes the multi-channel in situ monitoring signals obtained in the initial t time steps during the device fabrication process, where $s_{t}\in R^{4}$ consists of the diffuse reflectance signal (ND) and three photoluminescence signals at different wavelengths (LP725, LP780, and SP775). The complete process signal $s_{1:T}$ can only be obtained after the preparation is completed. At the early stage, only the partial signal $s_{1:t_{obs}}$ can be observed by the model up to a certain time point $t_{\mathrm{obs}}<T$. In this context, the objective of this paper is to jointly model the key attributes of the device, given only early observation signals $s_{1:t_{obs}}$. Particularly, joint probability distribution is shown as follows:


(1) $$p\left(r,m,y|\;s_{1:t_{obs}}\right)$$

Here, r represents the precursor ratio, m denotes the solution concentration, y represents the final photoelectric conversion efficiency (PCE) of the device, and $s_{t_{obs+1}:T}$ denotes the unobserved subsequent process signals. This modeling objective enables the model to simultaneously achieve device performance prediction and uncertainty estimation at an early stage, providing a unified probabilistic inference framework for online process decision-making.

### 3.2. Input Representation and Early-Observation Masking Mechanism

To handle early observation signals of varying lengths within a unified model, we adopt a fixed-length sequence modeling approach. All samples are represented as multi-channel time series of length T, where each time step contains four-dimensional process signals: ND, LP725, LP780, and SP775.

For a given sample, only the in situ monitoring signals up to an early observation time point $t_{\mathrm{obs}}$ are available. Nevertheless, the input to the model retains a fixed length T, while observed and unobserved time steps are distinguished through a masking mechanism. Specifically, when $t\leq t_{\mathrm{obs}}$, the true observed signal is provided as input; when $t>t_{\mathrm{obs}}$, the corresponding time steps are zero-padded solely for computational alignment. Importantly, the model does not rely on zero-padding alone. A strict key-padding mask is applied in the self-attention mechanism of the Transformer encoder to explicitly block unobserved time steps. Masked positions are assigned −∞ before the softmax operation, resulting in zero attention weights so that they do not contribute to feature aggregation. This strategical design allows the model—with completely shared structure and parameters—to flexibly adapt to various early-observation scenarios (such as 20 s, 30 s, 60 s, 120 s, etc.) without requiring separate training for different observation times.

### 3.3. Multi-Task Transformer Model

**Figure 2:**
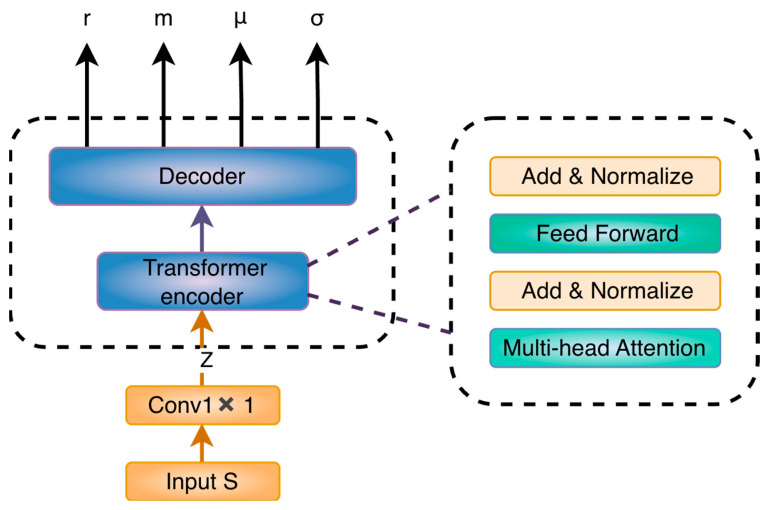
Architecture of the proposed multi-task Transformer for early-stage perovskite fabrication.

This paper proposes a Transformer-based multi-task encoder architecture that leverages a Transformer encoder to extract key temporal features from in situ detection signals during the early stages of perovskite fabrication, enabling accurate prediction of critical device properties. As illustrated in Figure 2, the proposed model consists of a shared Transformer encoder and multiple task-specific prediction heads, which jointly perform prediction tasks for device photoelectric conversion efficiency, solution concentration, and precursor ratio.

At the input stage, a one-dimensional convolution is first applied to the raw input sequence S to project it into a compact latent representation *Z*. This operation reduces the input dimensionality while capturing local temporal patterns, and the resulting representation is then fed into the Transformer encoder for global sequence modeling.


(2) $$Z={Conv}_{1\times 1}(S)\;$$

The latent representation *Z* is then fed into a Transformer encoder composed of a self-attention mechanism and a feed-forward neural network, following the standard architecture introduced in the original Transformer model by Vaswani et al. [36]. The encoder output is subsequently passed to a fully connected layer that serves as a decoder, which is responsible for producing the task-specific outputs, namely the precursor ratio r, solution concentration m, and the parameters μ and σ associated with the probabilistic prediction of device performance.

### 3.4. Probabilistic Modeling and Training Objectives

To characterize the uncertainty of different parameters involved in the perovskite fabrication process, this work adopts heterogeneous modeling strategies for different prediction targets within a unified multi-task learning framework.

The precursor ratio r and solution concentration m are discrete process parameters, whose values are selected from finite candidate sets and are explicitly defined during the experimental design stage. Accordingly, these two prediction tasks are formulated as multi-class classification problems. For each process parameter, the model outputs a probability distribution over all candidate classes, and the model parameters are optimized by minimizing the cross-entropy loss between the predicted distribution and the ground-truth category. Specifically, the classification losses corresponding to the precursor ratio and solution concentration are defined as


(3) $$L_{r}=-\sum_{k=1}^{7}y_{k}^{r}\log{p_{k}}^{r}\;$$

(4) $${\;L}_{m}=-\sum_{k=1}^{5}y_{k}^{m}\log{p_{k}}^{m}$$

where $y_{k}^{r}$ denotes the one-hot encoding of the ground-truth class for the precursor ratio, and ${p_{k}}^{r}$ represents the predicted class probability for the precursor ratio. Similarly, $y_{k}^{m}$ denotes the one-hot encoding of the ground-truth class for the solution concentration, and ${p_{k}}^{m}$ denotes the predicted class probability for the solution concentration.

The final device photoelectric conversion efficiency is treated as a continuous random variable, and is assumed to follow a Gaussian distribution conditioned on the early-stage observations and process conditions:

(5) $$p(y|s_{1:t_{\mathrm{obs}}})=N\left(\mu ,\sigma^{2}\right)\;$$

where y denotes the experimentally measured final photoelectric conversion efficiency of the device. After obtaining the predicted mean μ and standard deviation σ, the corresponding network parameters are learned via maximum likelihood estimation (MLE). Accordingly, the loss function for the PCE prediction task is constructed using the negative log-likelihood (NLL), which is defined as follows:


(6) $$L_{PCE}=-\log l\left(y|\mu ,\sigma \right)\;\;=-\log\left({\left(2\pi \sigma^{2}\right)}^{-1/2}\exp\left(-\frac{{\left(y-\mu \right)}^{2}}{2\sigma^{2}}\right)\right)\;=\frac{1}{2}\log\left(2\pi \right)+\log\left(\sigma \right)+\frac{{\left(y-\mu \right)}^{2}}{2\sigma^{2}}$$

μ is the predicted mean that reflects the expected device performance conditioned on the early-stage observations, and σ is the predicted standard deviation used to characterize the uncertainty of the prediction. By minimizing the loss function $L_{PCE}$, the model is able to capture predictive uncertainty while simultaneously fitting the conditional mean.

It should be clarified that the Gaussian assumption adopted in this study is intended as a conditional approximation rather than a strict assumption regarding the global distribution of PCE. In practical fabrication processes, PCE may exhibit skewness or heavy-tailed characteristics. The conditional Gaussian modeling employed here is primarily used to characterize uncertainty around the predicted mean, rather than to precisely describe the overall distributional shape.

The overall training objective is defined as a weighted sum of the losses associated with the individual tasks:

(7) $$L_{total}=\lambda_{PCE}L_{PCE}+\lambda_{r}L_{r}+\lambda_{m}L_{m}\;$$

where $L_{\mathrm{PCE}}$, $L_{r}$, and $L_{m}$ denote the previously defined loss functions for photoelectric conversion efficiency prediction, precursor ratio classification, and solution concentration classification, respectively. The non-negative coefficients $\lambda_{PCE}$, $\lambda_{r}$, and $\lambda_{m}$ are weighting factors that regulate the relative importance of different tasks within the multi-task learning framework.

### 3.5. Training and Inference Strategy

During training, a random early-observation strategy is adopted. Specifically, for each mini-batch, an observation time $t_{\mathrm{obs}}$ is randomly sampled from a predefined set of early observation times, and the corresponding input mask is constructed accordingly. The model performs multi-task prediction using only the partial observation $s_{1:t_{obs}}$, which enables it to progressively improve its inference capability as information accumulates during the learning process.

During inference, the input format remains identical to that used in the training stage. For any given early observation time $t_{\mathrm{obs}}$, the same trained model can directly output the predicted device performance along with its associated uncertainty, without requiring model switching or parameter adjustment. This unified training–inference mechanism ensures consistent inference behavior across different observation times and allows the model to flexibly adapt to various early-stage observation scenarios.

## 4. Experimental Results and Analysis

### 4.1. Dataset Description

To systematically evaluate the predictive capability of the proposed multi-task conditional Transformer model during the early phases of perovskite device fabrication, an in situ monitoring dataset derived from actual experimental processes has been employed. Each sample in the dataset corresponds to one complete perovskite thin-film fabrication experiment.

**Figure 3:**
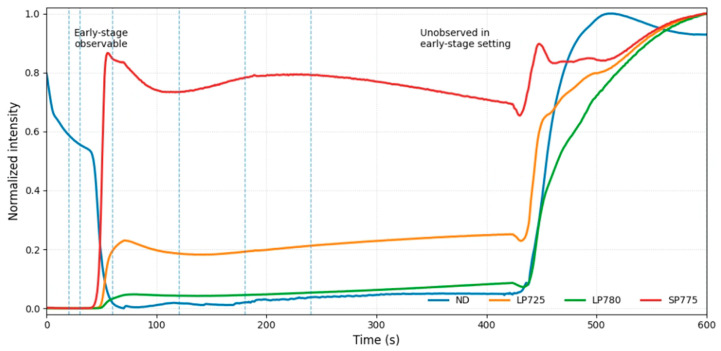
Representative in situ multi-channel optical signals during perovskite film formation.

During the fabrication process, a multi-channel time-series signal is continuously acquired through an in situ optical monitoring system to characterize the dynamic evolution behavior of the film formation stage. Specifically, diffuse reflection signals (ND) and three different bands of photoluminescence signals (LP725, LP780, and SP775) are synchronously recorded at each time step to characterize the crystallization quality, phase composition, and defect evolution behavior of the perovskite material. As shown in Figure 3, these in situ signals demonstrate distinct temporal evolution characteristics during the fabrication process.

Apart from process signals, the dataset also includes various label information closely related to the device fabrication pathway, e.g., precursor ratio, solution molarity, and the final device power conversion efficiency (PCE). Furthermore, the duration of vacuum quenching is recorded as a predefined process condition variable before the experiment begins. This study focuses on performance prediction at the early stages of fabrication. In the experimental setup, the model is only allowed to access the in situ monitoring signals from the initial few time steps of the fabrication process, corresponding to the early observation time points (20–600 s) marked by dashed lines in Figure 3. In short, the dataset shows high complexity in terms of signal dimensions, time scales, and prediction targets, providing a challenging experimental benchmark for validating the proposed method in this work.

### 4.2. Experimental Setup

The dataset was randomly divided at the sample level into a training set and a validation set, with 80% allocated for model training and the remaining 20% for validation. All in situ monitoring signals were uniformly normalized before being input into the model to scale and magnitude across different signal channels. For samples with inadequate actual monitoring duration, zero-padding was employed to unify the sequence length, and a masking mechanism was employed to ignore the corresponding invalid time steps in the model, thereby ensuring that this model only learns and makes predictions based on the actual observable signals.

To simulate real-world observation scenarios in the early stages of the preparation process, a random early-observation strategy was utilized during the training phase. In each training mini-batch, an observation time $t_{\mathrm{obs}}$ is randomly selected from a predefined set of early observation times, and only in situ monitoring signals before that time are provided to the model, while the remaining time steps are excluded via masking. The early observation time points selected for this study are 20 s, 30 s, 60 s, 120 s, 180 s, 240 s, and 600 s, corresponding to different physical stages during the fabrication process. The model is trained using a multi-task joint training strategy, with parameters updated via the AdamW optimizer. The initial learning rate is set to 1 × 10^−4^, and the training stability is improved by weight decay along with gradient clipping. The loss functions for different tasks are optimized by weighted combination, where the weights are tuned on the validation set. During the process of inference, the input format remains consistent with that in training. For any given early observation time, the same model can directly output the corresponding prediction results without the need for structural adjustments or model switching.

### 4.3. Evaluation Metrics

To quantitatively assess the model performance across different prediction tasks, differentiated evaluation metrics have been adopted. We take prediction accuracy, physical interpretability, and robustness to experimental noise into account within the selected joint indicators, thereby comprehensively reflecting the model’s capability in material- and device-level modeling.

Regarding the material composition identification task, classification accuracy is adopted as the primary evaluation metric and supplemented by confusion matrix analysis to test the model’s discriminative ability across different parameter levels. In addition, a confusion matrix is employed for fine-grained analysis of the classification results, facilitating the evaluation of the model’s discrimination stability and potential confusion patterns when distinguishing between similar parameter levels (such as molar ratios and solution concentrations). The above analysis is appropriate for revealing the model’s robustness and performance in handling subtle differences in material composition. For the device performance prediction task, the mean absolute error (MAE) is employed here, as the core evaluation metric to quantitatively characterize the deviation between the predicted photoelectric conversion efficiency (PCE) and the experimentally measured values. The MAE directly reflects the physical magnitude of the prediction error, providing good interpretability with insensitivity to outliers compared to mean squared error-based metrics. The above merits make it particularly suitable for horizontal comparisons among different models in device performance prediction tasks. Furthermore, to comprehensively assess the model’s predictive capability, this paper supplements the analysis with statistical metrics such as the coefficient of determination (R^2^), which measures the extent to which the model explains the variance in PCE.

In addition, to evaluate the model’s capability in modeling the dynamic evolution patterns of the preparation process, the reconstruction error has been calculated in unobserved time intervals within the task of process signal completion, thereby quantifying the model’s ability to recover the temporal structure of in situ monitoring signals. This metric indicates whether the model can implicitly learn the intrinsic kinetic patterns during the process of perovskite thin-film formation, even in the absence of partially observed information, thereby providing support for subsequent early-stage prediction and uncertainty analysis. Aided by the above multi-dimensional evaluation framework, we can systematically elucidate the comprehensive performance of different models across tasks such as material composition identification, device performance prediction, and preparation process modeling, offering unified and reliable quantitative evidence for subsequent model comparison and methodological improvement.

### 4.4. Early-Stage Identification of Material Composition and Process Parameters

During the fabrication of perovskite devices, the precursor material ratio and solution molar concentration are crucial process parameters that determine film formation quality and final device performance. If these parameters can be reliably identified in the early stages of fabrication based solely on partial in situ monitoring signals, it will provide fundamental support for process monitoring, anomaly detection, and experimental screening. This section evaluates the performance of the proposed multi-task conditional Transformer model in the task of early-stage identification of material composition and process parameters.

**Figure 4:**
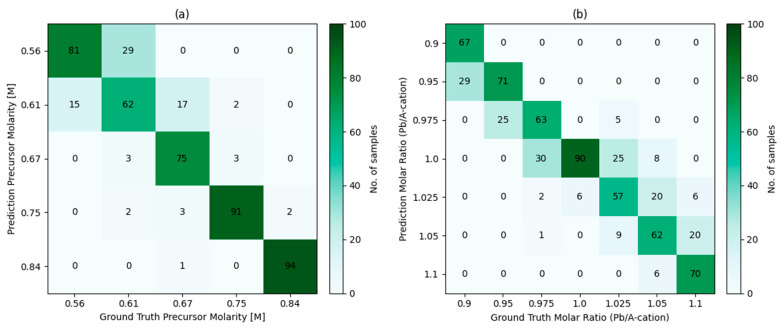
Early-stage identification of material composition and process parameters using in situ optical signals. (**a**) Confusion matrix for precursor molarity classification. (**b**) Confusion matrix for Pb/A-cation molar ratio classification.

Figure 4 displays the confusion matrix results of the model for the tasks of precursor molar concentration identification and molar ratio identification. Regarding the precursor molar concentration identification task, the model achieved a classification accuracy of 0.84 on the test set. When evaluated using the top-2 metric, its recognition accuracy further improved to 0.98. For the molar ratio (Pb/A-cation ratio) identification task, the model attained a classification accuracy of 0.71, with a corresponding top-2 accuracy of 0.94. Overall, the prediction results for both tasks exhibit a clear dominance along the main diagonal, suggesting that the model can effectively distinguish between different material parameter conditions at the early stages of preparation.

Further analysis of the confusion matrix indicates that misclassified samples are principally concentrated between parameter categories with close numerical values, whereas almost no confusion occurs under conditions with larger parameter differences. This phenomenon is physically reasonable: when the differences in molar concentration or stoichiometric ratio are small, their impacts on the optical responses (e.g., photoluminescence and diffuse reflectance signals) during the initial film formation stage are relatively limited, leading to high similarity in time-resolved features across different categories. As the parameter deviation increases, its impact on crystallization kinetics, phase evolution, and defect formation processes gradually strengthens, which is responsible for more pronounced and distinguishable features in the in situ monitoring signals.

The above results indicate that, even if based solely on partial time-resolved in situ monitoring signals acquired during the early stages of preparation, the model is capable of efficiently identifying key material compositions and process parameters, implying that rich information regarding material states is already embedded in the early dynamic signals. The extraordinary top-2 accuracy indicates that the model can consistently constrain the range of true parameters within a narrow interval of uncertainty, demonstrating strong early-stage discriminative ability. This not only validates the effectiveness of the model in capturing temporal features and performing representation learning but also offers a physically consistent and information-rich intermediate representation for subsequent tasks in device performance prediction. Thus, a coherent modeling pathway has been established between material formation dynamics and the final device performance.

### 4.5. Device Performance Prediction and Uncertainty Analysis Under Fixed Early Observation

During the fabrication process of perovskite devices, the final power conversion efficiency (PCE) can only be obtained after the fabrication process is fully completed [43,44]. However, in practical experiments and potential online decision-making scenarios, the ability to reliably predict device performance at early fabrication stages would facilitate the early identification of potentially defective samples, optimize the allocation of experimental resources, and significantly improve the overall production efficiency. Although current research has demonstrated that deep learning models can partially enable early performance prediction based on in situ monitoring data during fabrication, most of these approaches rely on traditional feed-forward or conventional deep learning (DL) models When processing time-resolved in situ signals, such models typically rely on fixed-length feature compression or local temporal modeling for prediction, thereby exhibiting limited capacity to capture long-range temporal dependencies. Furthermore, current methods primarily focus on point prediction accuracy, while lacking systematic characterization of prediction reliability and uncertainty, which heavily constrains their practical value in real-world process decision-making.

Based on the aforementioned background, this section systematically analyzes the capabilities of the proposed multi-task conditional Transformer model for device performance prediction and its uncertainty modeling under more rigorous and challenging conditions—specifically with observations fixed at an early time point—and conducts a comparative analysis against traditional deep learning models. To ensure comparability of results across different methods, a unified early-observation time window is selected as the input condition. Only in situ monitoring signals generated during the initial fabrication stage are provided to the models. Under identical data partitioning and input settings, the Transformer model and the baseline deep learning model are trained separately to predict the final device’s power conversion efficiency (PCE), with model prediction performance quantitatively assessed utilizing the mean absolute error (MAE), mean squared error (MSE), and the coefficient of determination (R^2^).

**Figure 5:**
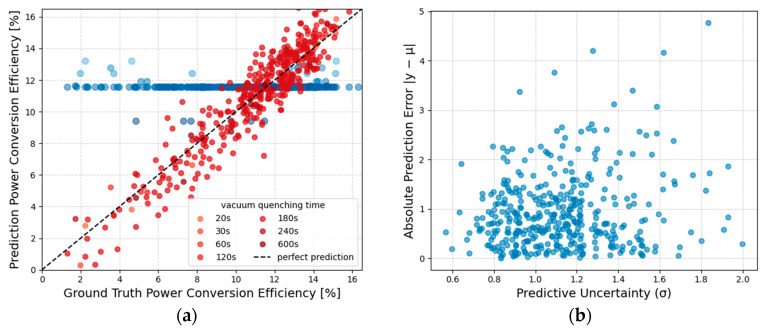
Early-stage device performance prediction and uncertainty analysis under fixed observation conditions. (**a**) Comparison between predicted and experimentally measured power conversion efficiency (PCE) values for different models using only early-stage in situ monitoring signals. The blue dots represent the baseline samples without vacuum quenching treatment. (**b**) Relationship between predictive uncertainty (σ) and absolute prediction error |y − μ| for the proposed Transformer model.

The comparative analysis presented in Figure 5a between the predicted PCE values from different models and the actual measured values under fixed early-stage observation conditions indicates that compared to the conventional DL model, the proposed Transformer model demonstrates significant advantages in both overall prediction accuracy and stability. Even when relying only on partial time-resolved in situ dynamic signals from the early fabrication stage, the predictions of the Transformer model remain more consistent with the actual PCE, indicating its ability to more effectively capture key information related to final device performance from early-stage temporal signals. Further quantitative evaluation is summarized in Table 1. Compared with the conventional DL model and baseline methods, the Transformer model significantly reduces MAE and MSE while achieving a higher R^2^ value across the entire test set as well as different data subsets.

On the contrary, although traditional DL models have significantly improved prediction performance compared to baseline methods, they still present relatively large prediction errors under fixed early-stage observation conditions, along with limited prediction stability across different samples. This difference primarily originates from the distinct model architectures: feed-forward DL models tend to rely more heavily on local feature representations during early stages, while the Transformer, through its self-attention mechanism, can adaptively assign weights across the entire observation time window. This enables it to simultaneously focus on early transient features and key signal changes that evolve over time, thereby providing an advantage in early prediction tasks with limited information availability.

Apart from point prediction performance, this study further provides a systematic assessment of the uncertainty characteristics in predictions from different models. Traditional deep learning (DL) models typically output only a single predicted value, posing substantial obstacles to provide a reliable measure of confidence for the predictions. In contrast, the proposed Transformer model, operating within a probabilistic modeling framework, can output the corresponding PCE prediction distribution given early observational signals and process conditions, thereby offering a quantitative description of confidence intervals for the predictions. Figure 5b illustrates the relationship between the prediction uncertainty of the Transformer model and its prediction errors. A significant correlation between the two can be observed: when prediction uncertainty is low, the corresponding prediction errors are generally small and tightly distributed, whereas, with respect to samples with high prediction uncertainty, the dispersion of prediction errors increases markedly. This phenomenon is not evident in the prediction results of traditional DL models, revealing that the uncertainty estimates provided by the Transformer exhibit higher consistency and discriminative capability in reflecting prediction reliability.

Further elucidation of the evolution of prediction uncertainty in the Transformer model over observation time indicates that during the very early stages of fabrication, due to the highly limited observable information, the model predictions exhibit an overall high level of uncertainty. As observation time gradually increases, the predictive distribution progressively converges, with uncertainty levels decreasing significantly and the model’s confidence in its predictions correspondingly rising. The decreasing uncertainty tendency with increasing observation time aligns with the evolution of prediction errors over time, further validating the physical plausibility of the Transformer under the framework of temporal modeling and probabilistic prediction.

## 5. Conclusions

To address the substantial process variability and the lack of reliable real-time predictive assessment mechanisms during thin-film formation, we propose a multi-task conditional Transformer framework based on machine learning (ML). The proposed approach is motivated by the observation that dynamic optical signals captured during the early stages of film formation encode sufficient information to forecast final process outcomes, provided that early kinetics can be effectively related to final properties under varying process conditions. Our primary contribution is a unified probabilistic modeling framework that jointly infers material composition parameters (precursor ratio and solution molarity), predicts power conversion efficiency (PCE), and captures the underlying process dynamics. By incorporating the evacuation duration as a prior condition and employing a shared Transformer encoder with a tailored early-observation masking mechanism, this design enables a single, robust model to operate consistently across multiple stages of the fabrication timeline, thereby providing probabilistic support for early-stage process assessment. The experimental results clearly demonstrate the effectiveness of the proposed framework. The final performance of perovskite devices can be reliably predicted before the completion of the fabrication process. Compared with conventional deep learning baselines, the proposed model achieves consistently higher prediction accuracy and stability, thereby enabling timely and informed process evaluation. In summary, the proposed Transformer-based architecture establishes an advanced deep learning model capable of handling partial observations while integrating process conditions, thereby unlocking the predictive potential embedded in in situ monitoring data. This capability provides a probabilistic modeling foundation for future closed-loop manufacturing systems aimed at enhancing yield and accelerating perovskite photovoltaic development. Future work will focus on validating this framework under practical manufacturing settings.

## Figures and Tables

**Table 1:** Quantitative comparison of early-stage PCE prediction performance under fixed observation conditions.

Metric		Entire Test Set	Subsets of Different Vacuum Quenching Times						
			20 s	30 s	60 s	120 s	180 s	240 s	600 s
MAE	Baselines	2.55	2.95	3.10	2.43	3.30	2.05	2.28	3.06
	DLmodel	1.44	1.93	1.25	1.35	1.21	1.38	1.56	1.59
	Transformer	1.32	1.71	1.16	1.22	1.12	1.27	1.42	1.45
MSE	Baselines	11.51	14.92	15.36	9.71	17.9	7.13	11.08	16.18
	DLmodel	4.29	7.80	2.92	3.29	2.69	4.04	4.72	5.26
	Transformer	3.85	6.41	2.46	2.90	2.30	3.36	3.90	4.60
$R^{2}$	Baselines	−0.01	0.00	−0.01	−0.04	−0.01	0.00	−0.02	−1.35
	DLmodel	0.62	0.12	0.54	0.55	0.72	0.48	0.43	0.38
	Transformer	0.68	0.28	0.59	0.61	0.77	0.55	0.51	0.46

## Data Availability

The original contributions presented in this study are included in the article. Further inquiries can be directed to the corresponding author.
